# Characterization and Transcriptional Regulation of *n*-Alkane Hydroxylase Gene Cluster of *Rhodococcus jostii* RHA1

**DOI:** 10.3390/microorganisms7110479

**Published:** 2019-10-23

**Authors:** Namiko Gibu, Daisuke Kasai, Takumi Ikawa, Emiko Akiyama, Masao Fukuda

**Affiliations:** Department of Bioengineering, Nagaoka University of Technology, Nagaoka 940-2188, Japan

**Keywords:** *n*-alkane, *n*-alkane hydroxylase, *Rhodococcus*, TetR-type transcriptional regulator

## Abstract

Gram-positive actinomycete *Rhodococcus jostii* RHA1 is able to grow on C10 to C19 *n*-alkanes as a sole source of carbon and energy. To clarify, the *n*-alkane utilization pathway—a cluster of 5 genes (*alkBrubA1A2BalkU*) which appeared to be involved in *n*-alkane degradation—was identified and the transcriptional regulation of these genes was characterized. Reverse transcription-PCR analyses revealed that these genes constituted an operon and were transcribed in the presence of *n*-alkane. Inactivation of *alkB* led to the absence of the ability to utilize *n*-undecane. The *alkB* mutation resulted in reduction of growth rates on C10 and C12 *n*-alkanes; however, growths on C13 to C19 *n*-alkanes were not affected by this mutation. These results suggested that *alkB* was essential for the utilization of C10 to C12 *n*-alkanes. Inactivation of *alkU* showed the constitutive expression of *alkB*. Purified AlkU is able to bind to the putative promoter region of *alkB*, suggesting that AlkU played a role in repression of the transcription of *alk* operon. The results of this study indicated that *alkB* was involved in the medium-chain *n*-alkanes degradation of strain RHA1 and the transcription of *alk* operon was negatively regulated by *alkU*-encoded regulator. This report is important to understand the *n*-alkane degradation pathway of *R. jostii*, including the transcriptional regulation of *alk* gene cluster.

## 1. Introduction

**Figure 1:**
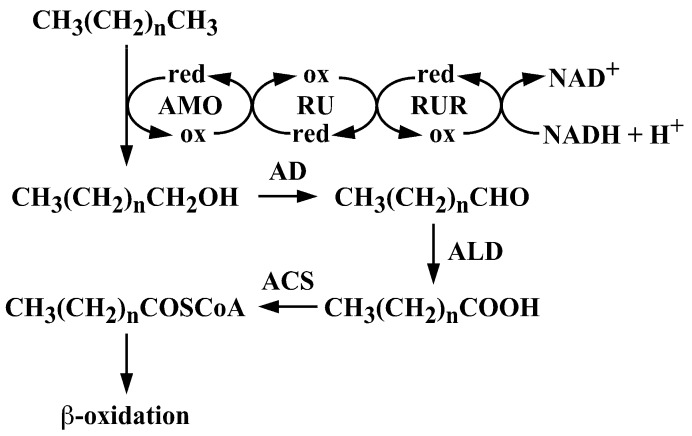
Aerobic pathways for the *n*-alkane degradation by terminal oxidation. Abbreviations: AMO, *n*-alkane 1-monooxygenase; RU, rubredoxin; RUR, rubredoxin reductase; AD, alcohol dehydrogenase; ALD, aldehyde dehydrogenase; ACS, acyl-CoA synthetase; ox, oxidized; and red, reduced.

Alkanes constitute up to 50% of crude oil and are commonly found in oil-contaminated environments [1]. Various microorganisms, both aerobic and anaerobic, utilize alkanes as a sole carbon and energy source [2,3]. Aerobic degradation of *n*-alkane is usually initiated by the alkane hydroxylase, which converts alkanes to alkanols [4,5,6]. The resulting alkanols are oxidized by the sequential reactions of alcohol and aldehyde dehydrogenases to yield the corresponding fatty acids, which are further metabolized by β-oxidation after conjugation of coenzyme A by an acyl CoA synthetase (Figure 1) [2,3,7]. The alkane-hydroxylation mechanism of *Pseudomonas putida* GPo1 has been characterized in detail, and consists of three subunits: an integral-membrane non-heme di-iron monooxygenase encoded by *alkB*; a rubredoxin encoded by duplicated genes, *alkF* and *alkG*; and a rubredoxin reductase encoded by *alkT* [8]. These genes are distributed in two different loci of the OCT plasmid in the strain GPo1 [8]. Their transcription is regulated by LuxR/UhpA-like transcriptional regulator encoded by *alkS* [8]. In the case of *Acinetobacter baylyi* ADP1, a three-component alkane hydroxylase containing alkane 1-monooxygenase encoded by *alkM*, a rubredoxin encoded by *rubA*, and a rubredoxin reductase encoded by *rubB*, which is similar to the GPo1 enzyme, catalyzes terminal alkane oxidation [9,10]. Transcription of *alkM* depends strictly on AraC/XylS-like transcriptional activator encoded by *alkR* and is induced by alkanes of various chain lengths [11].

Other types of alkane-hydroxylation mechanism have also been reported. A flavin-binding monooxygenase encoded by *almA* is responsible for oxidation of C32 and C36 *n*-alkanes in *Acinetobacter* sp. DSM17874 [12]. In *Geobacillus thermodenitrificans* NG80-2, a soluble monomeric monooxygenase encoded by *ladA*, is involved in oxidation of C15 to C36 *n*-alkanes [13]. CYP153-family cytochrome P450 monooxygenases have been reported to catalyze terminal *n*-alkane oxidation in *Acinetobacter* sp. EB104, *Mycobacterium* sp. HXN-1500, and *Alcanivorax dieselolei* B-5 [5,14,15].

Mycolic acid-containing actinomycetes, including *Rhodococcus*, have been increasingly recognized as candidates for the biodegradation of hydrocarbons because of their capacity to degrade a wide range of organic compounds and to produce biosurfactants, hydrophobic cell surface, and ubiquity and robustness in the environment [16]. Recently, *alkB* homologs, which codes for alkane 1-monooxygenase, have been characterized in actinomycetes, including *Rhodococcus opacus* B-4 [17], *Rhodococcus ruber* SP2B [18], *Rhodococcus* sp. BCP1 [19], *Rhodococcus* sp. TMP2 [20], *Rhodococcus* sp. NRRL B-16531 [21], *Rhodococcus erythropolis* Q15 [21], *Gordonia* sp. SoCg [22,23], and *Nocardioides* sp. CF8 [4]. However, little is known about the catabolic operon structure and regulatory mechanism of *n*-alkane degradation genes in actinomycetes. In this study, *n*-alkane degradation genes in *Rhodococcus jostii* RHA1 were examined. Strain RHA1 was isolated from a lindane-contaminated soil, and it co-metabolizes polychlorinated biphenyls with biphenyl via the biphenyl metabolic pathway [24,25]. It utilizes a wide range of hydrocarbons—such as aromatic compounds, propane, and steroids—as a sole source of carbon and energy [26,27,28,29]. In this study, we found that RHA1 utilized C10 to C19 *n*-alkanes, and characterized the operon structure and transcriptional regulation of *n*-alkane degradation genes in RHA1.

## 2. Materials and Methods

### 2.1. Bacterial Strains, Plasmids, and Culture Conditions

The bacterial strains and plasmids used in this study are listed in Table 1. *Rhodococcus jostii* RHA1 and its mutant derivatives were routinely grown at 30 °C in Luria-Bertani (LB) medium, 0.2× LB medium, or W minimal salt medium [30] containing 10 or 20 mM *n*-alkanes (C9 to C30) or 20 mM pyruvate. In solid cultures on W minimal medium agar, *n*-alkanes (C5 to C13) were supplemented as vapor. *Escherichia coli* strains were grown in LB medium at 37 °C. For cultures of cells carrying antibiotic resistance markers, the media for *E. coli* transformants were supplemented with 100 mg of ampicillin/liter or 25 mg of kanamycin/liter.

### 2.2. DNA Manipulations, Nucleotide Sequencing, and Sequence Analysis

Total DNA isolation and electroporation were performed as described in a previous study [37]. DNA sequencing was performed by the Eurofins sequencing service (Eurofins Genomics, Tokyo, Japan). Analysis of nucleotide sequences and homology searches were carried out as previously described [38]. The genome sequence of RHA1, whose accession numbers are NC_008268, NC_008269, NC_008270, and NC_008271, was used to find *n*-alkane catabolic genes at the RHA1 genome database (http://www.rhodococcus.ca/index.jsp).

### 2.3. Construction of Disruption Mutants

The *alkB* and *alkU* genes were separately disrupted using the *sacB* counterselection system essentially as described previously [29,35,39]. Oligonucleotides that amplified flanking regions of each gene were listed in Table 2. Disruption of the genes was examined by PCR analysis. Disruption of the genes was confirmed by diagnostic PCR using specific primer sets and subsequently by DNA sequencing of the PCR amplified regions flanking the deletions.

### 2.4. Growth Curves on n-Alkanes

RHA1 and its mutant derivatives were grown in 10 mL of 0.2× LB medium for 36 h at 30 °C. Cells were harvested and washed three times with W medium and suspended in the same medium. Afterwards, the bacterial suspension was inoculated in 50 mL of W medium supplemented with 10 or 20 mM *n*-alkanes. The cells were incubated at 30 °C under shaking conditions and at each time point 1-mL aliquots were sampled and centrifuged at 5000× *g* and the pellet was dried to a constant weight.

### 2.5. Resting Cell Assay

RHA1 and DAB were grown on 0.2× LB or W minimal medium containing 20 mM pyruvate or 10 mM *n*-alkanes to give an OD_600_ of 1.0. The cells were collected by centrifugation, washed twice with 2 mL of W medium, and resuspended in a same medium to give an OD_600_ of 1.0. One milliliter of cell suspension was preincubated for 5 min at 30 °C and was incubated in a sealed 4.5-mL glass vial with shaking at 30 °C after the addition of 1 mM of *n*-alkanes. Control cells were inactivated by being autoclaved at 121 °C for 15 min prior to the addition of substrate. To stop the reaction 0.1 mL of 6 N HCl was added to the mix and 1 mM of phenanthrene was added as an internal standard. After the addition of NaCl to saturation, 3 mL of ethyl acetate was added to the mixture, which was then mixed on a vortex mixer for 1 min. The supernatant was recovered, dehydrated with sodium sulfate, evaporated, and dissolved in 150 μL of ethyl acetate. One microliter of extract was analyzed by gas chromatography-mass spectrometry (GC-MS; model 5971A; Agilent Technologies Co., Palo Alto, CA, USA), equipped with an Ultra-2 capillary column (50 m by 0.2 mm; Agilent Technologies) as described previously [25].

### 2.6. RNA Isolation

RHA1 and *alkU*-deficient mutant (DAU) were grown at 30 °C in 100 mL of W medium containing 10 mM *n*-undecane or 20 mM pyruvate. The resulting cells were harvested by centrifugation, washed with RNA protect bacterial reagent (QIAGEN, Germantown, MD, USA), and stored at −80 °C. Total RNA extraction from frozen cells was performed as described previously [40]. Purified RNA was then treated with RNase-free DNase I (Takara Bio Inc., Otsu, Japan) to remove contaminating DNA.

### 2.7. Reverse Transcription (RT)-PCR and Quantitative RT-PCR (qRT-PCR)

Single-stranded cDNA was synthesized from 1.0 µg of total RNA utilizing ReverTra Ace reverse transcriptase (Toyobo, Osaka, Japan) with random primers (Invitrogen, Carlsbad, CA, USA) in a 20-µL reaction mixture. PCR amplification was performed using 2.0 µL of the cDNA mixture, specific primers (Table 2), and Ex *Taq* DNA polymerase (Takara Bio Inc.) under the following conditions: 95 °C for 30 s and 30 cycles of 95 °C for 30 s, 60 °C for 60 s, and 72 °C for 60 s. A control without reverse transcriptase was used for each reaction to verify the absence of genomic DNA contamination. Samples from the PCR were electrophoresed on a 2.0% agarose gel and visualized with ethidium bromide.

The amount of each transcript was measured using StepOne Plus real-time PCR system (Life Technologies, Carlsbad, CA, USA). Each primer set was designed using Primer Express version 3.0 software (Life Technologies). For qRT-PCR, 100 ng of cDNA sample, 100 nmol of each primer (Table 2), and 10 μL Fast SYBR Green PCR Master Mix (Life Technologies) were mixed in a final volume of 20 μL. The qRT-PCR was carried out using the following conditions: 95 °C for 20 sec and 40 cycles of 95 °C for 3 sec and 60 °C for 30 sec. The fluorescence signal from SYBR Green I intercalating into double-stranded DNA was detected during each annealing step. The copy number of each gene was determined based on the standard curve of the target gene, which was estimated by the results of qRT-PCR performed using 10-fold serial dilutions of each standard sample. To normalize the amount of RNA in each sample, 16S rRNA was used as an internal standard. Each measurement was carried out in triplicate, and the means and standard deviations were calculated.

### 2.8. Purification of His-Tagged alkU

The coding region of *alkU* was amplified by PCR using the specific primer pair (Table 2). The amplified fragment was cloned into NdeI-BamHI-digested pET16b by using In-Fusion cloning strategy [41]. The cells of *E. coli* BL21 (DE3) harboring the resultant plasmid (pETALKU) were grown at 30 °C. Expression of the gene was induced by adding 0.5 mM isopropyl-β-D-thiogalactopyranoside when the absorbance at 600 nm (*A*_600_) of the culture reached 0.5. After 4-h induction, the cells were harvested by centrifugation at 5000× *g* at 4 °C for 10 min and were resuspended in 50 mM Tris-HCl buffer (pH 7.5). The cells were then disrupted by an ultrasonic disintegrator (UD-201; Tomy Seiko Co., Tokyo, Japan) and centrifuged at 15,000× *g* at 4 °C for 15 min, to generate crude cell extract.

To remove nucleic acids, streptomycin sulfate was added to the crude extracts to a final concentration of 1%. The lysate was kept on ice for 10 min and centrifuged at 15,000× *g* for 15 min. The supernatant was applied to a Ni Sepharose 6 Fast Flow column (GE Healthcare, Buckinghamshire, UK) previously equilibrated with buffer A consisting of 50 mM Tris-HCl (pH 7.5), 500 mM NaCl, and 100 mM imidazole. Proteins were allowed to bind for 1 min at 4 °C while rotating, followed by washing five times in 5 mL of buffer A. His-tagged proteins were eluted with 5 mL of buffer B consisting of 50 mM Tris-HCl (pH 7.5), 500 mM NaCl, and 500 mM imidazole, and the fractions were pooled and concentrated.

### 2.9. Electrophoretic Mobility Shift Assays (EMSAs)

DNA fragments containing the upstream region of *alkB* were prepared by PCR with the specific primer pairs (Table 2). The 3′ ends of the probe fragments were labeled with DIG-11-ddUTP using the second-generation DIG gel shift kit (Roche), according to the manufacturer’s instructions. Binding reaction was performed at 20 °C for 20 min in a 10-μL reaction mixture containing 50 ng of the purified his-tagged AlkU (His-AlkU), 1 nM DIG-labeled probe, 1 μg of poly[d(I-C)], 0.1 μg of poly-L-lysine, 20 mM HEPES (pH 7.6), 1 mM EDTA, 10 mM (NH_4_)_2_SO_4_, 1 mM dithiothreitol, 0.2% (*w*/*v*) Tween 20, and 30 mM KCl. Gel electrophoresis and the detection of signals were performed as described previously [42].

## 3. Results

### 3.1. Growth of RHA1 on n-Alkanes

To determine growth characteristics of RHA1 on *n*-alkanes, RHA1 was grown in liquid W minimal medium containing 10 mM *n*-alkanes ranging in length from C9 to C30 and on W medium agar supplemented with C5 to C13 as vapor. RHA1 grew on C11 to C19 *n*-alkanes in liquid medium (Table 3). It did not grow on C9, C10, C20, and C30 *n*-alkanes in liquid medium. On agar medium, it grew on *n*-decane in addition to C11 to C13 *n*-alkanes. It did not grow on C5 to C9 *n*-alkanes on agar medium. Based on these results, we concluded that RHA1 utilized C10 to C19 *n*-alkanes as a sole source of carbon and energy.

### 3.2. The alk Gene Cluster of Strain RHA1

**Figure 2:**
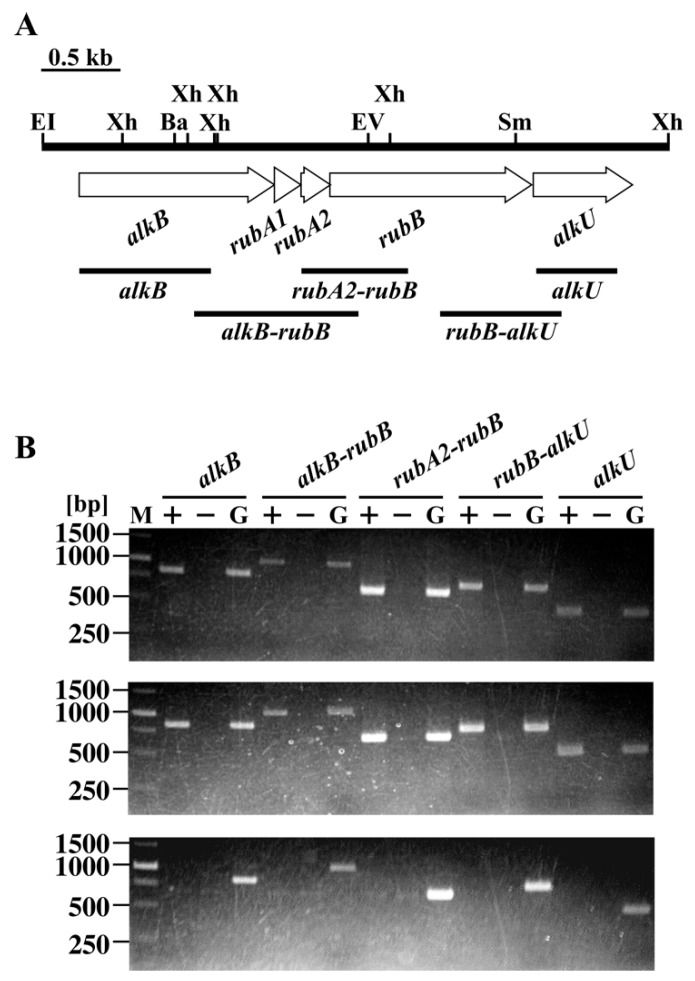
Organization and RT-PCR analysis of the *alk* gene cluster in RHA1. (**A**) Open arrows indicate the sizes, locations, and transcriptional directions of ORF. Boldfaced bars below the gene cluster diagram indicate the locations of the amplified RT-PCR products shown in panel B. Abbreviations for restriction enzymes: Ba, BamHI; EI, EcoRI; EV, EcoRV; Sm, SmaI; and Xh, XhoI. (**B**) Total RNA used for cDNA synthesis was isolated from RHA1 cells grown on *n*-undecane (**upper panel**), *n*-hexadecane (middle panel), and pyruvate (**lower panel**). Agarose gel electrophoresis of RT-PCR assays with primers targeting *alkB* (expected size, 831 bp), *alkB*-*rubA1-rubA2-rubB* (expected size, 1039 bp), *rubA2-rubB* (expected size, 671 bp), *rubB-alkU* (expected size, 766 bp), and *alkU* (expected size, 510 bp) are shown. Positions of amplified regions and primer sequences are indicated in panel A and Table 2, respectively. Lane M, molecular weight markers; Lanes + and −, RT-PCR with and without RT, respectively; lane G, control PCR with the RHA1 genomic DNA.

A BLASTp homology search using the amino acid sequence of alkane 1-monooxygenase (AlkB) from *R. opacus* B-4 (YP_002779449) as a query indicated that its closest ortholog is ro02534 in the genome database of RHA1. Hence, ro02534 was named as *alkB*. The region downstream of ro02534 contained orthologs of genes involved in *n*-alkane catabolism. Based on the amino acid sequence similarities, ro02535, ro02536, and ro02537 encode a couple of rubredoxins and a rubredoxin reductase, responsible for electron transfer in alkane hydroxylase [43], and were named as *rubA1*, *rubA2*, and *rubB*, respectively (Figure 2A). The ro02538 gene located next to *rubB* showed similarity to a putative TetR-type transcriptional regulator, and was named as *alkU*.

To examine operon structure of the *alk* gene cluster, RT-PCR analysis was performed with total RNA prepared from RHA1 cells grown on either *n*-undecane or pyruvate. Amplification products with the expected sizes of the segments *alkB*, *alkB-rubB*, *rubA2-rubB*, *rubB-alkU*, and *alkU* were detected using RNA prepared from the cells grown on *n*-undecane (Figure 2B). Based on these results, *alkB*, *rubA1*, *rubA2*, *rubB*, and *alkU* might be included in the same transcriptional unit. No amplification products were detected when RNA from pyruvate-grown cells was used, suggesting that transcription of these genes is induced after growth on *n*-undecane.

### 3.3. Disruption of alkB in RHA1

**Figure 3:**
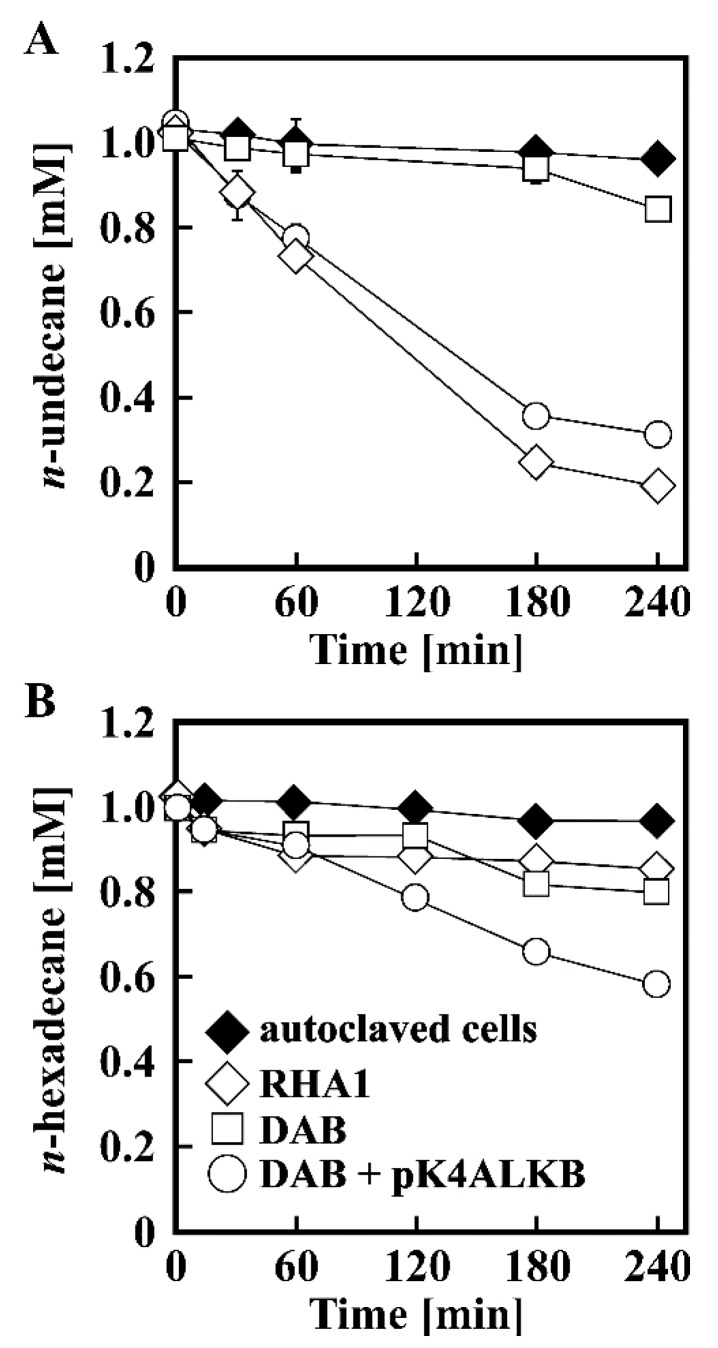
Degradation of *n*-undecane (**A**) and *n*-hexadecane (**B**) by RHA1, DAB, and DAB harboring pK4ALKB. 1 mM of each *n*-alkanes was incubated with the resting cells of RHA1 (open diamonds), DAB (open squares), DAB harboring pK4ALKB (open circles), and autoclaved cells of RHA1 (closed diamonds). The degradation activities were determined by GC-MS analysis. Each value is the average ± standard deviation of three independent experiments.

In order to evaluate the role of *alkB* in *n*-alkane utilization of RHA1, *alkB* was inactivated by gene replacement technique with the *alkB* disruption plasmid, pABS*mobsacB*. The degradation activities of the resting cells of RHA1 and the resulting mutant, DAB, toward *n*-undecane were determined by GC-MS. As shown in Figure 3A, DAB almost completely lost the activity to degrade *n*-undecane under the assay conditions used. To determine if this deficiency was caused by the disruption of *alkB*, a plasmid, pK4ALKB, containing an intact *alkB* was introduced into DAB to complement the mutation. Complementation with *alkB* restored these degradation activities to a level approximately equal to that of the wild-type strain (Figure 3A). Based on this result, we concluded that *alkB* was essential for degradation of *n*-undecane. In contrast, *n*-hexadecane degradation activity of DAB was almost the same as that of RHA1 (Figure 3B), suggesting that *alkB* was not essential for the utilization of *n*-hexadecane, and other gene(s) were involved in this degradation. However, the cells of DAB harboring pK4ALKB showed a higher activity than that of the wild-type strain. It is therefore probable that the *alkB* product has the ability to degrade *n*-hexadecane.

**Figure 4:**
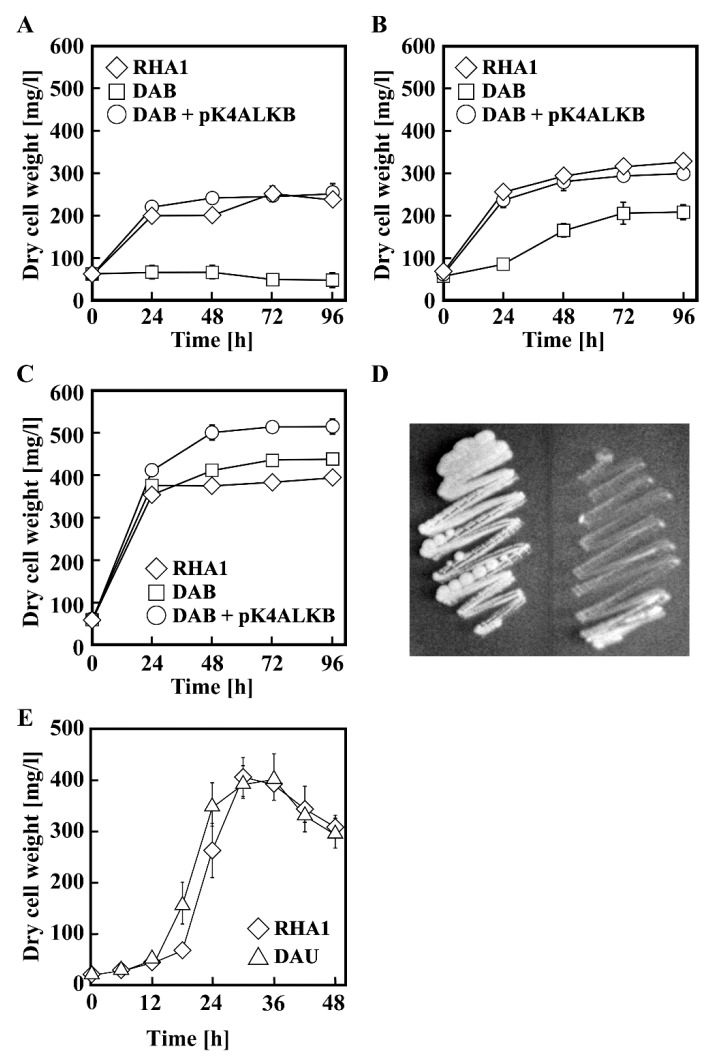
Growth of RHA1 and its mutant derivatives on *n*-undecane (**A**), *n*-dodecane (**B**), *n*-hexadecane (**C**), and *n*-decane (**D**). (**A**–**C**) RHA1 (diamonds), DAB (squares), DAB harboring pK4ALKB (circles) were grown in W medium containing 10 mM of each *n*-alkanes. Each value is the average ± standard deviation of three independent experiments. (**D**) Growth of RHA1 (left) and DAB (right) on *n*-decane after 5 days of incubation. (**E**) Growth of RHA1 (diamonds) and DAU (triangle) on 20 mM *n*-undecane.

To estimate the level of participation of *alkB* in the growth of RHA1 on *n*-alkane, the growth ability of DAB was compared to that of RHA1. The capacity of DAB to grow in both liquid and solid media containing *n*-undecane was completely lost (Figure 4 and Table 3). When the *alkB* mutation was complemented by the introduction of the corresponding gene in trans, the growth deficiency was restored. When DAB was grown in *n*-dodecane and *n*-decane, the growths decreased (Table 3). These results indicated that *alkB* is fundamentally engaged in the utilization of C10 to C12 *n*-alkanes. On the other hand, disruption of *alkB* did not affect the growth on *n*-alkane from C13 to C20 (Table 3), suggesting that other gene(s) were involved in the *n*-alkanes utilization.

### 3.4. Transcriptional Regulation of alk Operon

The *alkU* gene product (AlkU) is similar in sequence to putative transcriptional regulators belonging to the TetR family from *Rhodococcus* sp. Q15 (AAK97457) and *R. opacus* B-4 (BAH50508). To confirm that *alkU* gene product played a role in the transcriptional regulation of the *alk* operon, *alkU* was disrupted by homologous recombination. The growth ability on *n*-undecane of the resulting mutant strain DAU was slightly higher than that of the wild-type strain (Figure 4E). The results suggest that AlkU negatively regulated the transcription of *alk* genes and was responsible for the induction of these genes.

**Figure 5:**
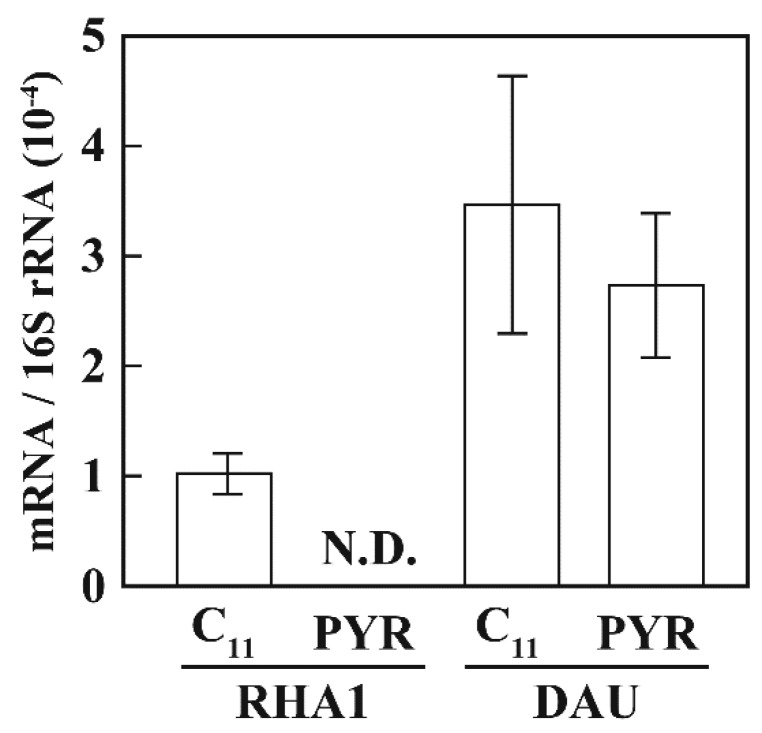
qRT-PCR analysis of the expression of *alkB* in RHA1 and DAU. The cells of RHA1 and DAU were grown in W medium containing 10 mM *n*-undecane (C11) or 20 mM pyruvate (PYR). Values for each mRNA expression were normalized to 16S rRNA gene expression. The data are mean values ± standard deviations for three independent experiments. N.D.: Not detected.

To examine AlkU function, the level of transcription of *alkB* in strain DAU was examined by quantitative RT-PCR. When the RHA1 cells grew on pyruvate, mRNAs of *alkB* was not detected in our analytical condition (Figure 5). On the other hand, the transcription was significantly increased in the cells grown on *n*-undecane, indicating that the transcription of the *alk* genes was induced during *n*-undecane utilization. In contrast, DAU cells showed constitutive expression of *alkB* (Figure 5). The results suggest that AlkU was essential for the transcriptional regulation of *alk* operon as a repressor.

**Figure 6:**
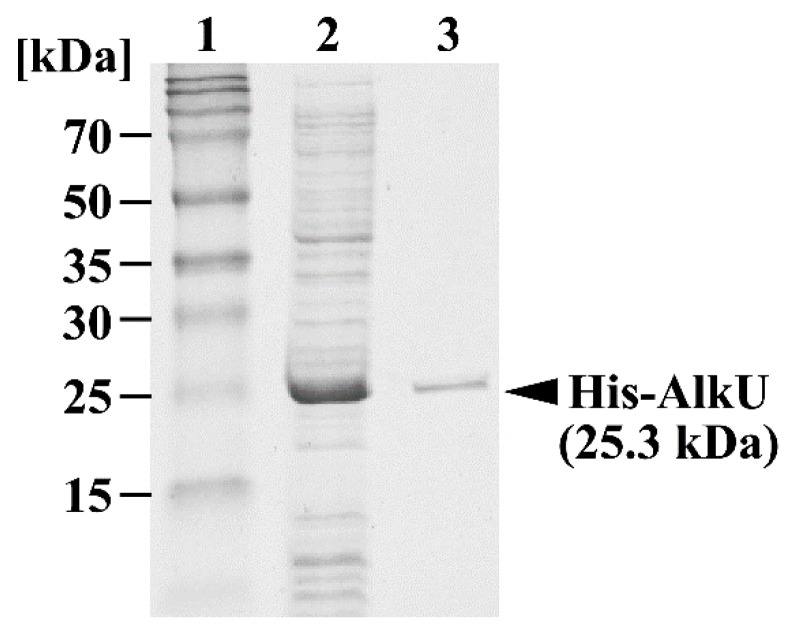
SDS-PAGE analysis of protein fraction. Lanes: 1, molecular-weight markers; 2, Crude cell extract of *E. coli* BL21 (DE3) harboring pETALKU (10 µg of protein); 3, Elution fraction including His-AlkU (2 µg of protein).

**Figure 7:**
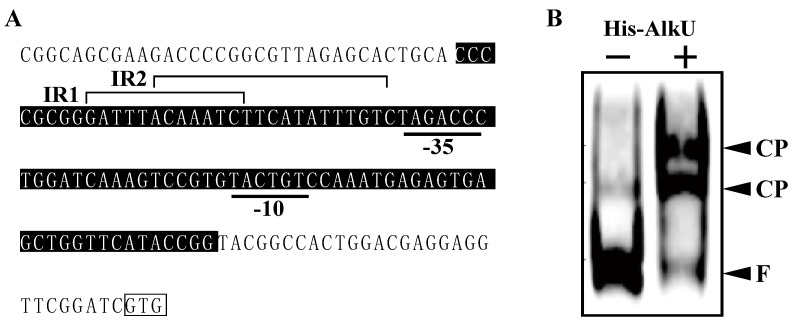
Binding of His-AlkU to the upstream region of *alkB*. (**A**) Schematic diagrams of the upstream region of *alkB*. Black boxes revealed the region of DNA probe used in EMSAs. Underlines indicate a putative -10 and -35 sequences. Open box indicates start codon of *alkB*. Putative IR sequences are shown on top of nucleotide sequences. (**B**) EMSAs of the binding of purified His-AlkU (50 ng) to the DNA probe. The positions of the free probe and His-AlkU-DNA complex are shown by F and CP, respectively. + and − indicate presence and absence of His-AlkU, respectively.

His-tag fused *alkU* was expressed in *E. coli* BL21 (DE3) cells to produce His-tagged AlkU (His-AlkU). SDS-PAGE analysis revealed the expression of a 25.3-kDa protein, consistent with the deduced amino acid sequence (Figure 6). Purified His-AlkU was used in electrophoretic mobility shift assays (EMSAs) with DNA probes containing the upstream region of *alkB* (Figure 7A). His-AlkU bound this DNA region (Figure 7B), indicating that the direct binding of AlkU with the promoter region of the *alk* operon.

## 4. Discussion

In this study, we identified and characterized the *alkB* gene and its transcriptional regulation in *R. jostii* RHA1, which could grow on *n*-alkanes ranging in length from C10 to C19 as the sole source of carbon and energy. Though RHA1 grows on propane [29], this strain is unable to utilize C5 to C9 *n*-alkanes. Similar observations have been reported for *R. opacus* B-4 and *Rhodococcus* sp. BCP1, which are unable to grow on C5 to C6 and C8 to C11 *n*-alkanes, respectively [17,19]. These observations may indicate that the hydroxylases for C5 to C9 *n*-alkanes degradation are absent in RHA1.

In the deduced amino acid sequence of *alkB*, the conserved eight-histidine motif thought to be required for catalytic activity of non-heme di-iron integral membrane alkane hydroxylases was detected [44,45]. Kyte-Doolittle hydropathy analysis of the amino acid sequence deduced from AlkB of RHA1 showed similarity to those of other *Rhodococcus* strains and *P. putida* GPo1 [46], suggesting that AlkB of RHA1 was membrane bound (data not shown). AlkB of GPo1 oxidizes *n*-alkanes limited to C13 and has a bulky amino acid-like tryptophan at position 55 [47]. On the other hand, alkane 1-monooxygenase from *Rhodococcus* sp. strain NRRL B-16531, *R. erythropolis* Q15, and *Mycobacterium tuberculosis* H37Rv, with valine or leucine residue at the corresponding position, were shown to oxidize *n*-alkanes up to at least C16 [21,47]. Since leucine residue was found at the position of AlkB from RHA1, and the *n*-hexadecane degradation activity increased by introduction of *alkB* on a multicopy-number plasmid, this enzyme might be responsible for the long-chain *n*-alkane degradation. However, the growth on *n*-alkanes from C13 to C19 was not affected by deletion of *alkB*, whereas the *alkB* transcription was observed during the growth with *n*-hexadecane. It is suggested that *alkB* is a minor contributor to C13 to C19 *n*-alkanes utilization, and unidentified gene(s) played a major role in the degradation of these *n*-alkanes.

AlkM from *Acinetobacter* sp. strain ADP1 oxidizes C12 to C18 *n*-alkanes [11]. *Acinetobacter* sp. strain M-1 was shown to possess two alkane monooxygenases encoded by *alkMa* and *alkMb*, involved in the degradation of up to C16 and C30 *n*-alkanes, respectively [48]. In addition, AlmA from *Acinetobacter* sp. strain DSM 17874 [12], LadA from *G. thermodenitrificans* NG80-2 [13], and cytochrome P450 monooxygenases [5,14,15] were reported to be involved in long-chain *n*-alkane degradation. In silico mining of the RHA1 genome database revealed the genes encoding for five AlmA homologs, a LadA homolog, and 31 putative cytochrome P450 monooxygenases. According to the deduced amino acid sequence similarity, some of these genes seemed to be responsible for C13 to C19 *n*-alkane utilization in RHA1.

The organization of *alk* genes is similar to that of the genes from several *Rhodococcus* strains [17,19,21], demonstrating that the gene clusters appear to have a common ancestral origin. RT-PCR analysis revealed that *alkB*, *rubA1*, *rubA2*, *rubB*, and *alkU* are transcribed as an operon (Figure 2B). The transcripts of these genes were observed in cells growing on both *n*-undecane and *n*-hexadecane, suggesting that the transcription of the *alk* operon was induced by these *n*-alkanes or their metabolites. In qRT-PCR analysis revealed the transcriptional induction of *alk* operon during the growth on *n*-undecane (Figure 5). The operon was constitutively transcribed in the absence of *alkU*. Furthermore, the EMSA revealed that AlkU bound the upstream region of *alkB* (Figure 7). These results strongly suggested that *alkU* codes for a repressor of the *alk* operon. In several Gram-negative bacteria, the transcriptions of the *alk* genes are under the control of a LuxR-type transcriptional regulator, AlkS binding the inverted repeat (IR) sequences located in directly upstream of the -35 region of *alkB* and *alkS* promoters [8,49,50]. In contrast, AlkU belongs to the TetR family of transcriptional regulators that regulate the transcription of genes for antibiotic resistance, antibiotic biosynthesis, catabolic pathway, and biofilm formation in Gram-negative and Gram-positive bacteria [51]. TetR-type transcriptional regulators are known to act both as a repressor and an activator [51,52]. TetR, which is involved in the transcriptional repression of tetracycline resistance gene, *tetA*, is known to act as a homodimer and binds the palindromic operator overlapping with the *tetA* and *tetR* promoters [53,54]. Furthermore, LuxR of *Vibrio harveyi* activates and represses the transcription of quorum-sensing genes by binding palindromic sequences located upstream or downstream of the promoter regions [52]. In the case of the *alkB* promoter region of *Rhodococcus* strains—including RHA1, BCP1, and B-4—two IR sequences, IR1 (GATTTacAAATC) and IR2 (ACAAATcTtcAtATTTGT), are well conserved [17,19], suggesting that AlkU may recognize to the IR sequences, in order to control the transcription of the *alk* operon. Since two different AlkU-DNA complexes were observed, AlkU may interact with multiple binding sites in the promoter in the same manner as other TetR family proteins [55]. The IR2 sequence is located immediately upstream of a putative -35 (TAGACC) sequence. Therefore, the transcription of *alk* genes appeared to be inhibited by the AlkU binding to the promoter region. It has been reported that the transcription of *alkB* of BCP1 and B-4 is also activated in the presence of *n*-alkanes [17,19]. The transcriptional regulation of the *alk* genes from these *Rhodococcus* stains was similar to that from RHA1.

In this study, we identified the *alk* operon involved in the medium-chain *n*-alkanes degradation of strain RHA1. According to the mutant analysis of *alkB*, the gene is required for the growth on C10 to C12 *n*-alkanes. The *alkU* gene encodes a transcriptional repressor of *alk* operon, and *n*-alkanes act as inducers. It is necessary to identify the long-chain *n*-alkane oxidation genes in order to gain a better understanding of *n*-alkane degradation by this strain.

## Figures and Tables

**Table 1:** Strains and plasmids used in this study.

Strain or Plasmid	Relevant Characteristic(s) ^a^	Source or Reference
Strains		
*R. jostii*		
RHA1	Wild type	[25]
DAB	RHA1 derivative; Δ*alkB*	This study
DAU	RHA1 derivative; Δ*alkU*	This study
*E. coli*		
JM109	*endA1 recA1 gyrA96 thi hsdR17 relA1 supE44* Δ(*lac-proAB*) *mcrA* [F’, *traD36 proAB*^+^ *lacl*^q^ Δ*ZM15*]	[31]
S17-1	RK2 *tra* regulon, λ *pir*, host for *pir*-dependent plasmids	[32]
BL21(DE3)	F^−^ *ompT hsdS*_B_(r_B_^-^m_B_^-^) *gal dcm* (DE3); T7 RNA polymerase gene under the control of the *lacUV*5 promoter	[33]
Plasmids		
pT7Blue	Cloning vector; T7 promoter, Ap ^r^	Novagen
pBluescript II KS(+)	Cloning vector; Ap ^r^	[34]
pK19*mobsacB*	*oriT sacB* Km ^r^	[35]
pK4	*Rhodococcus*-*E. coli* shuttle vector, Km ^r^	[36]
pET16b	Expression vector, N-terminal His_10_ tag, Ap ^r^ T7 promoter	Novagen
pTALKBL	pT7Blue with a 917-bp PCR fragment generated by alkBL_F/R primer pair	This study
pTALKBR	pT7Blue with a 723-bp PCR fragment generated by alkBR_F/R primer pair	This study
pTALKBLR	pTALKBR with a 733-bp EcoRI-SacI fragment of pTALKBL	This study
pABS*mobsacB*	pK19*mobsacB* with a 1.5-kb EcoRI-HindIII fragment of pTALKBLR	This study
pTALKBD	pT7Blue with a 967-bp PCR fragment generated by alkBD_F/R primer pair	This study
pT7BL	pT7Blue with a 0.8-kb EcoRI-BamHI fragment of pTALKBL	This study
pT7B	pT7BL with a 0.7-kb BamHI-HindIII fragment of pTALKBD	This study
pK4ALKB	pK4 with a 1.5-kb EcoRI fragment of pT7B	This study
pTUPU	pT7Blue with a 761-bp PCR fragment generated by UPalkU_F/R primer pair	This study
pTDWU	pT7Blue with a 792-bp PCR fragment generated by DWalkU_F/R primer pair	This study
pKU	pK19*mobsacB* with a 1.6-kb HindIII fragment carrying part of *alkU*	This study
pETALKU	pET16b with a 0.7-kb PCR amplified fragment carrying of *alkU*	This study

^a^ Abbreviations; Ap ^r^ and Km ^r^, resistance to ampicillin and kanamycin, respectively.

**Table 2:** Primer sequences used in this study.

Primer	Sequence (5′ to 3′) ^a^
alkB_F	TGACGACGTCGAATATCAGC
alkB_R	CCTGAATGATCAGGAACGG
alkBrubB_F	GAGCATTCACAACGATGTGC
alkBrubB_R	ACAGGAAGTCCTTCGACACC
rubA2B_F	GTACCGATTTCAAGCTCTACC
rubA2B_R	CACATCCGATGAGACCTCC
rubBalkU_F	ACGGTCGAAGTTGGAGTGC
rubBalkU_R	CTTTGTAGATCGTCTGCCTGC
alkU_F	CGAGCGACAGAGTCGATACC
alkU_R	ATGAAACTCAAGGCGAGCC
alkBL_F	ACAGGTGAAGCTACCAGCG
alkBL_R	GTGAGAGCTTCTGGACTTTCC
alkBR_F	CTTCGGTGAGAGCTTCTGG
alkBR_R	AAGCTTGTCGTCGGGCACATCG (HindIII)
alkBD_F	AACAGCTCGAGAACGACAGG
alkBD_R	GAATTCCATCACCGAACTCCGC (EcoRI)
UPalkU_F	AAGCTTTGTCCTCGCTCGACGTGAGC (HindIII)
UPalkU_R	GGATCCATTGTCGGCCAGGGACGTTCG (BamHI)
DWalkU_F	GGATCCAACGGTCGTGGGTGAACTCG (BamHI)
DWalkU_R	AAGCTTAGGAACTGATCTACGCCAACC (HindIII)
HISalkU_F	TCGAAGGTCGTCATAGCAGACGACCGCACCGCAGACGACCGCACC
HISalkU_R	GGATCCTCGAGCATAAAGTACTCACGGGTGAAGTACTCACGGGTG

^a^ Engineered restriction sites are underlined, and the corresponding restriction enzymes are shown in parentheses.

**Table 3:** Growth of *R. jostii* RHA1 and DAB on *n*-alkanes.

Media	Strains	*n*-Alkanes *^a^*																
		C5	C6	C7	C8	C9	C10	C11	C12	C13	C14	C15	C16	C17	C18	C19	C20	C30
Liquid	RHA1	ND	ND	ND	ND	−	−	+	+	+	+	+	+	+	+	+	−	−
	DAB	ND	ND	ND	ND	ND	−	−	±	+	+	+	+	+	+	+	−	−
Solid	RHA1	−	−	−	−	−	+	+	+	+	ND	ND	ND	ND	ND	ND	ND	ND
	DAB	ND	ND	ND	ND	ND	±	−	±	+	ND	ND	ND	ND	ND	ND	ND	ND

*^a^* ND, not determined. “+”, “–” and “± indicate significant, weak, and no growth, respectively.
